# Unraveling the link between malnutrition and adverse clinical outcomes: association of acute and chronic malnutrition measures with blood biomarkers from different pathophysiological systems

**DOI:** 10.1186/cc14473

**Published:** 2015-03-16

**Authors:** S Felder, N Braun, A Kutz, M Batschwaroff, P Schuetz

**Affiliations:** 1 Kantonsspital Aarau, Switzerland

## Introduction

Malnutrition is common in hospitalized medical patients and is associated with poor clinical outcomes. Whether malnutrition has a direct link to adverse outcomes or is rather a mirror of the severe patient condition remains debated. Our aim was to study the association of acute and chronic malnutrition status with blood biomarkers from different pathophysiological concepts to better understand the underlying mechanisms of malnutrition.

## Methods

We prospectively followed consecutive adult medical inpatients hospitalized between February 2013 and October 2013 in a tertiary care Swiss hospital. Nutritional risk was assessed using the Nutritional Risk Screening (NRS 2002) score, which incorporates acute and chronic measures of malnutrition. Multiadjusted regression models were used to investigate the associations between acute and chronic nutritional risk and biomarkers mirroring inflammation (CRP, PCT, proADM, leucocytes), stress (copeptin), renal dysfunction (creatinine, urea), nutritional status (vitamin D25, albumin, calcium, glucose), and hematological function (platelets, INR, Hb, RDW). Biomarker levels were transformed into deciles due to skewed distributions.

## Results

A total of 529 patients (mean age 72 years, 57.1% male) were included. Overall, there was a significant association of NRS and most biomarkers of inflammation, stress, renal function, nutrition and the hematological system (coefficient and 95% CI): CRP 0.021, *P *= 0.0021, PCT 0.28, *P *= 0.003, proADM 0.4, *P *< 0.001, copeptin 0.44, *P *< 0.001, urea 0.28, *P *= 0.002, vitamin D25 -0.23, *P *= 0.012, albumin -0.6, *P *< 0.001, hemoglobin -0.5, *P *< 0.001, RDW 0.46, *P *< 0.001. These associations remained robust after adjustment for sociodemographics (model 1), comorbidities (model 2) and main medical diagnosis (model 3). Subgroup analysis suggested that mainly the acute part of malnutrition and not chronic malnutrition was associated with an increase in biomarker levels.

## Conclusion

Acute malnutrition was associated with a pronounced inflammatory response and an increase in biomarkers from different pathophysiological systems which may partly explain the link between malnutrition and adverse medical outcomes. However, interventional trials are needed to prove causal relationships.

